# Are Hypometric Anticipatory Postural Adjustments Contributing to Freezing of Gait in Parkinson’s Disease?

**DOI:** 10.3389/fnagi.2018.00036

**Published:** 2018-02-15

**Authors:** Christian Schlenstedt, Martina Mancini, Jay Nutt, Amie P. Hiller, Walter Maetzler, Günther Deuschl, Fay Horak

**Affiliations:** ^1^Department of Neurology, University Hospital Schleswig-Holstein, Christian-Albrechts-University, Kiel, Germany; ^2^Balance Disorders Laboratory, Oregon Health & Science University, Portland, OR, United States

**Keywords:** postural control, anticipatory postural adjustment, postural balance, posture, Parkinson’s disease, freezing of gait, electromyography

## Abstract

**Introduction:** This study aims at investigating whether impaired anticipatory postural adjustments (APA) during gait initiation contribute to the occurrence of freezing of gait (FOG) or whether altered APAs compensate for FOG in Parkinson’s disease (PD).

**Methods:** Gait initiation after 30 s quiet stance was analyzed without and with a cognitive dual task (DT) in 33 PD subjects with FOG (PD+FOG), 30 PD subjects without FOG (PD-FOG), and 32 healthy controls (HC). APAs were characterized with inertial sensors and muscle activity of the tensor fasciae latae (TFL), gastrocnemius, and tibialis anterior was captured with electromyography recordings. Nine trials (of 190) were associated with start hesitation/FOG and analyzed separately.

**Results:** PD+FOG and PD-FOG did not differ in disease duration, disease severity, age, or gender. PD+FOG had significantly smaller medio-lateral (ML) and anterio-posterior APAs compared to PD-FOG (DT, *p* < 0.05). PD+FOG had more co-contraction of left and right TFL during APAs compared to PD-FOG (*p* < 0.01). Within the PD+FOG, the ML size of APA (DT) was positively correlated with the severity of FOG history (NFOG-Q), with larger APAs associated with worse FOG (rho = 0.477, *p* = 0.025). ML APAs were larger during trials with observed FOG compared to trials of PD+FOG without FOG.

**Conclusions:** People with PD who have a history of FOG have smaller ML APAs (weight shifting) during gait initiation compared to PD-FOG and HC. However, start hesitation (FOG) is *not* caused by an inability to sufficiently displace the center of mass toward the stance leg because APAs were larger during trials with observed FOG. We speculate that reducing the acceleration of the body center of mass with hip abductor co-contraction for APAs might be a compensatory strategy in PD+FOG, to address postural control deficits and enable step initiation.

## Introduction

Start hesitation or gait initiation failure, the inability to successfully transition from a standing posture to walking, is a common motor impairment in Parkinson’s disease (PD) and can result in falls (Bloem et al., 2004; Latt et al., 2009). “Start hesitation” is included in the broader definition of FOG, reported as a “brief, episodic absence or marked reduction of forward progression of the feet despite the intention to walk” (Giladi and Nieuwboer, 2008). The transition between an upright static posture and walking is characterized by a shift of the center of foot pressure posteriorly and laterally toward the stepping leg to accelerate the center of mass forward and laterally toward the stance leg to unload the stepping leg, called an anticipatory postural adjustment (APA) (Halliday et al., 1998). It is known that people with PD show hypometric and longer APAs during self-initiated gait, as well as a reduced first step speed and longer step latency compared to HC (Burleigh-Jacobs et al., 1997; Halliday et al., 1998; Rocchi et al., 2006; Mancini et al., 2009; Rogers et al., 2011). Furthermore, the size of APAs is dependent on initial stance width (Rocchi et al., 2006) and on the specific stepping condition (i.e., voluntary versus compensatory or cued stepping) (Schlenstedt et al., 2017).

The pathophysiology of start hesitation is not fully understood. It has been shown that people with PD who have FOG (PD+FOG) have impaired postural control compared to individuals without FOG (PD-FOG) (Duncan et al., 2015; Schlenstedt et al., 2016; Bekkers et al., 2017). Therefore, start hesitation might be the consequence of impaired weight-shifting, resulting in inability to unload the stepping leg. One study showed reduced ML APAs in PD+FOG compared to PD-FOG during cued step initiation in the on medication state, supporting the idea that weight-shifting might be compromised in PD+FOG (Tard et al., 2014). In contrast, other studies did not find any differences between PD+FOG with PD-FOG in the on medication state in ML size of APAs during self-initiated and cued step initiation (Delval et al., 2014; Plate et al., 2016; de Souza Fortaleza et al., 2017). Similar size AP APAs have been found for voluntary step initiation (Delval et al., 2014) whereas smaller AP APAs have been found for cued step initiation (Delval et al., 2014; Tard et al., 2014) in PD+FOG compared to PD-FOG in the on medication state. Furthermore, inconsistent results have been reported for the number of APAs (Delval et al., 2014; Plate et al., 2016; Cohen et al., 2017). Few studies found that PD+FOG more often perform multiple APAs prior to self-initiated (Delval et al., 2014) and compensatory stepping than PD-FOG (medication on and off) (Jacobs et al., 2009). In contrast, other studies did not report any differences between PD+FOG and PD-FOG in number of APAs during self-initiated (medication on) (Plate et al., 2016) and cued stepping (medication on and off) (Delval et al., 2014; Plate et al., 2016; Cohen et al., 2017).

In contrast to the notion that start hesitation originates from reduced weight-shifting, the initial APA weight shift might be unimpaired in PD+FOG but they may have difficulty coupling APAs with the stepping pattern (Nutt et al., 2011). Impaired posture–gait coupling is supported by Jacobs et al. (2009) who suggested that knee trembling during FOG episodes represents multiple APAs that are poorly coupled to a step. This study compared PD+FOG with HC and further research is necessary comparing PD+FOG with PD-FOG.

It has been shown that PD+FOG have impaired cognitive function compared to PD-FOG (Peterson et al., 2016; Yao et al., 2017). When performing a cognitive (Vercruysse et al., 2012) and motor task simultaneously, PD+FOG have larger dual task (DT) cost compared to PD-FOG during gait and gait initiation (Camicioli et al., 1998; Spildooren et al., 2010; de Souza Fortaleza et al., 2017). FOG is also more often elicited during dual tasking than single task stepping (Giladi and Hausdorff, 2006; Rahman et al., 2008; Heremans et al., 2013). Studying step initiation with a DT may better distinguish between PD+FOG and PD-FOG and to elicit FOG episodes, which is difficult to provoke in a laboratory setting.

If PD+FOG have impaired APAs it is not known whether altered APAs are a cause or consequence of FOG, i.e., are they more or less impaired during an actual freezing event. Most of the studies were conducted in the on medication state, with fewer, if any, trials with FOG (Schaafsma et al., 2003). It has been shown that levodopa increases the size and decreases the duration of APAs so we study subjects with PD in the off medication state (Burleigh-Jacobs et al., 1997; Curtze et al., 2015).

We propose two hypotheses: Start hesitation might be caused by insufficient ML weight shifting in the APA. Alternatively, the APAs may be altered to compensate for FOG. The aim of this study was to objectively characterize APAs during self-initiated gait, with and without a DT, in subjects with PD who have a history of FOG, and in trials with observed freezing, compared to matched subjects with PD without FOG and HC.

## Materials and Methods

### Participants

Thirty-three PD+FOG, 30 PD-FOG, and 32 HC participated in this study. Subjects with PD were recruited from the Neurology Department of Oregon Health & Science University, Portland, OR, United States. Participants with PD were diagnosed according to Brain Bank Criteria for PD (Gelb et al., 1999). The following exclusion criteria were applied: any other neurological disorders other than PD, deep brain stimulation, and orthopedic impairments that interfere with gait or balance. Participants were considered to be PD+FOG if they answered “yes” in item 1 of the NFOG-Q (Nieuwboer et al., 2009). FOG and its subtypes were carefully explained with video examples before completing the NFOG-Q. Trials with FOG (start hesitation) were identified by video by blinded investigators.

Balance was assessed with the Mini-Balance Evaluation Systems Test (Mini-BESTest) (Franchignoni et al., 2010; Schlenstedt et al., 2015). Cognition was assessed with the Montreal Cognitive Assessment (MoCA). Disease severity was assessed with the Unified PD Rating Scale (MDS-UPDRS) Part III (Goetz et al., 2008).

Parkinson’s disease groups were well balanced for disease duration and motor symptom severity, and all groups were fairly balanced for age (**Table 1**). This study was carried out in accordance with the recommendations of the local ethics committee with written informed consent from all subjects. All subjects gave written informed consent in accordance with the Declaration of Helsinki. The protocol was approved by the local ethics committee.

**Table 1:** Participant characteristics (*n* = 95).

Variable	PD+FOG (*n* = 33)	PD-FOG (n = 30)	HC (*n* = 32)	p-value
Age (years)	69.2 (6.5)	69.6 (8.5)	69.4 (6.8)	0.970
Gender (M/F)	25/8	20/10	18/14	0.250^#^
BMI (kg/m^2^)	26.3 (6.4)	27.0 (5.2)	25.4 (3.9)	0.503
Disease duration (years)	7.8 (5.1)	6.7 (5.1)	n/a	0.400
H&Y stage	2.5 (0.8)	2.2 (0.5)	n/a	0.065
MDS-UPDRS III	44.2 (13.2)	41.1 (10.1)	n/a	0.300
PIGD	6.4 (3.4)	4.8 (2.7)	n/a	0.043^∗^
Mini-BESTest	16.6 (6.0)^a^	19.1 (4.1)^b^	23.9 (2.6)^a,b^	<0.001^∗∗^
MoCA	24.4 (3.9)^c^	25.5 (3.5)	26.8 (2.9)^c^	0.013^∗^
NFOG-Q	13.8 (4.8)	n/a	n/a	n/a


*Values are mean (SD) or number of participants; p-value of one-way ANOVA, independent samples T-test or ^*#*^Chi-square test. ^*a,b,c*^Significantly different (p < 0.01); ^∗^Significantly different (p < 0.05); ^∗∗^Significantly different (p < 0.01).*

### Testing Procedure

Participants were asked to voluntarily start walking at a comfortable pace after 30 s of quiet standing. Feet position was standardized for all participants with a template [feet externally rotated and heel-to-heel distance fixed at 10 cm (Mancini et al., 2016)], which was removed before starting each trial. Stance and gait initiation were assessed without (single task) and with a cognitive DT (serial subtraction by 3s). PD subjects were assessed in the morning, the OFF state of medication, i.e., after withdrawal from all anti-parkinsonian medication for at least 12 h.

Kinematic data were collected from three inertial sensors (Opals by APDM, Inc.) worn on the posterior trunk at the level of L5, and on the right and left lower shins. Validity of the assessment of APAs via inertial sensors has been proofed previously (Mancini et al., 2009; Mancini et al., 2016). Muscle activity of the right and left TFL, TIB, and GAS was measured by surface electromyography (EMG, Wave by Cometa). The two systems, Mobility Lab with Opals and EMG, were synchronized.

### Assessment of Start Hesitation

To assess whether the participants showed start hesitation while initiating gait, all trials were videotaped and videos were rated by two independent raters: an expert neurologist in movement disorders (AH) and a movement scientist (CS). Raters were blinded to group allocation and participants were rated according to FOG criteria described by Schaafsma et al. (2003).

### Data Analysis

#### Inertial Sensors

**FIGURE 1:**
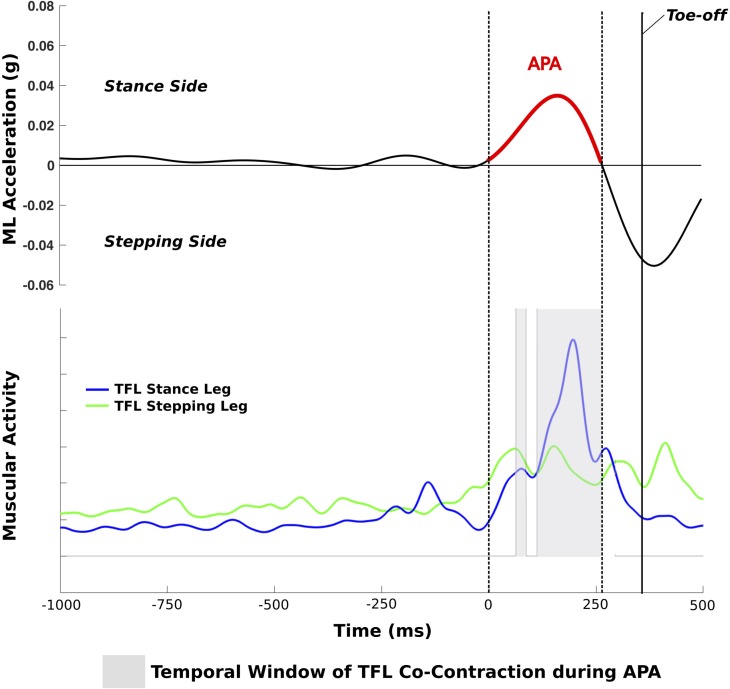
Trial of a PD+FOG with a single APA with TFL co-contraction during the APA; dotted lines represent the start and end of the APA.

**FIGURE 2:**
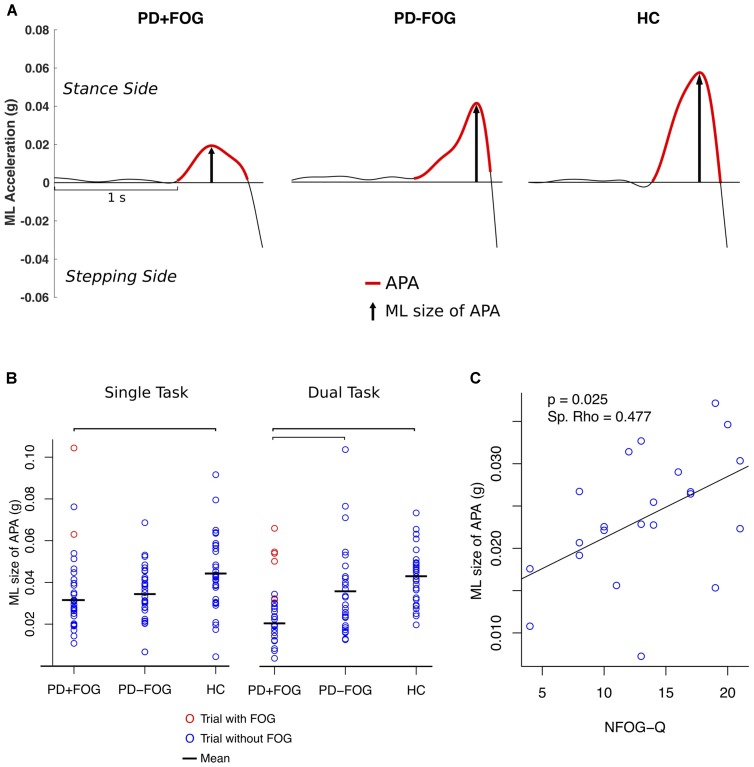
**(A)** Example of a trial of a PD subject with FOG (PD+FOG), without FOG (PD–FOG) and a HC. **(B)** Single subject plot of ML size of APA; bars indicate significant differences between the means (*p* < 0.01, Holm–Bonferroni adjusted). **(C)** Spearman’s rank correlation between the ML size of APA (DT condition) and NFOG-Q.

Postural sway during 30 s of quiet stance and APAs prior to gait initiation were quantified by the trunk Opal, whereas the first step characteristics were assessed with the Opals on the shins. The sampling frequency of the Opals was 128 Hz. Inertial data were filtered with a fourth-order low pass Butterworth filter with a cut-off frequency of 3 Hz. APAs and step kinematics were calculated by algorithms previously described (Mancini et al., 2016). In brief, a shift of the center of body mass toward the stance leg was considered to be an APA if the ML trunk acceleration exceeded 3 SD of baseline postural sway, calculated over the last 5 s of quiet stance. Start and end of an APA was defined as the moment when the signal crossed 1 SD of baseline sway prior to/after an APA. The following variables were calculated: (1) ML and AP size of APA: ML/AP peak acceleration from baseline sway as illustrated by examples in **Figure 1**; (2) APA duration: time from start to end of APA; (3) step latency: time from start of APA to toe off; (4) number of APAs; (5) first step shin range of motion (as a proxy for first step length); (6) first step time (as a proxy for first step velocity) (Mancini et al., 2016); and (7) gait speed (APDM Saw). To relate the size of a ML APA to the amount of postural sway during quiet stance, the following sway measures were calculated from the acceleration signal over 30 s: (8) ML sway length; (9) ML RMS as a measure of sway dispersion; (10) ML sway mean velocity; (11) ML sway frequency as the frequency comprising the 95% of the power (Mancini et al., 2011; Mancini et al., 2012). Matlab (R2016a) was used for data analysis.

#### EMG Analysis

The standard ISEK guidelines for surface EMG data acquisition and collection were followed (Merletti, 2017). Surface EMG data were recorded with a sampling frequency of 2000 Hz, full-wave rectified, and low-pass filtered with a second-order Butterworth filter with a cut-off frequency of 20 Hz. A muscle was considered to be active if the EMG signal exceeded 10% of maximal EMG activity. The following EMG variables were computed: (i) co-contraction: amount of time the following two muscles were simultaneously active during APA (%): left and right TFL, GAS and TIB of the stance leg, and GAS and TIB of the stepping leg (**Figure 1**); (ii) duration of activity of the TFL, GAS, and TIB of the stance and stepping leg, respectively, during APA. The inertial sensor and EMG data of each trial were graphically displayed and visually checked for artifacts and algorithm detection of events.

### Statistical Analysis

Participant characteristics were analyzed with a one-way ANOVA (except for gender: Chi-square test) or with an independent samples *T*-test in cases of only two groups. A natural logarithmic transformation was applied if data were not normally distributed. A 3 × 2 mixed measures ANOVA was used to investigate the effects of groups (PD+FOG, PD-FOG, HC), condition (single task and DT), and interaction. *Post hoc* comparisons were conducted with Holm–Bonferroni adjustment for multiple comparisons (Holm, 1979). Trials with FOG were excluded from the statistical analysis. The number of APAs was analyzed with a Chi-square test. As EMG data were not normally distributed a Kruskal–Wallis test was conducted with Mann–Whitney *U*-tests and Wilcoxon signed-rank test for *post hoc* comparisons (Holm–Bonferroni adjustment for multiple comparisons). Spearman’s rank correlation was computed to relate the ML size of APA with different outcome variables. The pre-defined level of significance was set at *p* < 0.05. Statistical analysis was performed with R (version 1.0.136) (R Development Core Team, 2010).

## Results

PD+FOG and PD-FOG had similar disease duration (*p* = 0.4) and disease severity (H&Y: *p* = 0.065; MDS-UPDRS III: *p* = 0.3). PD+FOG showed worse postural instability and gait difficulty (PIGD) subscore (*p* = 0.043) than PD-FOG and PD+FOG performed worse than HC in the MoCA (*p* < 0.01). The three groups did not differ in age (*p* = 0.97) or gender (*p* = 0.25). **Table 1** summarizes the participants’ characteristics.

### Subjects with a History of FOG Showed Smaller APAs Compared to the Other Groups

A significant group effect was found for the ML size of APA (*F* = 10.9; *p* < 0.001) (**Table 2**). *Post hoc* comparisons showed that PD+FOG had significantly smaller ML APAs compared to HC (*F* = 7.3; *p* = 0.034) in the single task condition and significantly smaller ML APA than both the PD-FOG (*F* = 11.7; *p* = 0.005) and HC (*F* = 26.0; *p* < 0.0001) in the DT condition (**Figures 2A,B**). A significant group effect was also found for AP size of APA (*F* = 4.6; *p* = 0.013) with a significant difference between PD+FOG and PD-FOG in DT condition (*F* = 9.9; *p* = 0.014). No significant differences between groups were found for APA duration or step latency.

**Table 2:** Results of the 3 × 2 ANOVA of APA characteristics, first step kinematics, gait speed, and postural sway measures.

					Group effect		Condition effect		Interaction effect	
							
Variable	Condition	PD+FOG	PD-FOG	HC	*F*	*p*-value	*F*	*p*-value	*F*	*p*-value
ML size of APA (g)	Single task	0.032 (0.014)	0.034 (0.013)	0.044 (0.018)	10.900	<0.001^∗∗^	0.262	0.610	2.825	0.066
	Dual task	0.024 (0.008)^a,b^	0.038 (0.021)^a^	0.045 (0.013)^b^						
AP size of APA (g)	Single task	0.032 (0.018)	0.042 (0.022)	0.041 (0.023)	4.600	0.013^∗^	0.889	0.349	0.828	0.441
	Dual task	0.027 (0.015)^c^	0.043 (0.025)^c^	0.040 (0.015)						
APA duration (s)	Single task	0.68 (0.49)	0.74 (0.57)	0.73 (0.79)	0.628	0.537	0.393	0.532	0.086	0.918
	Dual task	0.61 (0.22)	0.64 (0.33)	0.55 (0.20)						
Latency (s)	Single task	0.79 (0.51)	0.87 (0.58)	0.83 (0.78)	1.925	0.153	0.004	0.947	0.342	0.711
	Dual task	0.78 (0.25)	0.78 (0.33)	0.64 (0.17)						
First step range of motion (°)	Single task	27.3 (8.6)^d,e^	33.4 (6.2)^d^	40.7 (5.9)^d,g^	23.256	<0.001^∗∗^	15.661	<0.001^∗∗^	1.452	0.241
	Dual task	26.1 (8.9)^e,f^	33.0 (6.2)^f^	38.5 (5.1)^f,g^						
First step time (s)	Single task	0.36 (0.10)	0.41 (0.12)	0.36 (0.10)	0.886	0.416	2.663	0.107	0.809	0.449
	Dual task	0.43 (0.16)	0.41 (0.18)	0.38 (0.09)						
Gait speed (m/s)	Single task	0.87 (0.19)^h,l^	0.93 (0.15)^i,m^	1.12 (0.14)^h,i,n^	15.521	<0.001^∗∗^	113.894	<0.001^∗∗^	0.803	0.452
	Dual task	0.76 (0.18)^j,l^	0.82 (0.15)^k,m^	0.97 (0.14)^j,k,n^						
Postural sway length ML (m/s^2^)	Single task	3.77 (2.36)^o^	3.79 (2.07)^p^	3.07 (1.23)^q^	0.128	0.880	66.357	<0.001^∗∗^	9.826	<0.001^∗^
	Dual task	4.39 (1.90)^o^	4.65 (2.50)^p^	6.33 (3.81)^q^						
Postural sway RMS ML (m/s^2^)	Single task	0.023 (0.015)	0.024 (0.011)^r^	0.024 (0.014)^s^	0.949	0.392	31.939	<0.001^∗∗^	2.357	0.102
	Dual task	0.024 (0.009)	0.033 (0.020)^r^	0.033 (0.021)^s^						
Postural sway MV ML (m/s)	Single task	0.036 (0.038)	0.032 (0.023)	0.034 (0.033)	1.352	0.265	5.517	0.021^∗^	0.993	0.375
	Dual task	0.031 (0.023)	0.047 (0.037)	0.035 (0.030)						


*Values represent mean (SD). ^∗^Significantly different (p < 0.05); ^∗∗^Significantly different (p < 0.001); significant post hoc comparisons (Holm–Bonferroni adjusted for multiple comparisons): ^*c,g*^p < 0.05; ^*a,d,e,f,o,p,r*^p < 0.01; ^*i*^p < 0.001; ^*b,h,j,k,l,m,n,q,s*^p < 0.0001*.

The number of APAs was similar in all three groups for the single task condition (*p* = 0.335; number of participants with no/one/multiple APA: PD+FOG: 2/24/6, PD-FOG: 0/23/7, HC:0/27/5) and for the DT condition (*p* = 0.485; PD+FOG: 1/26/3, PD-FOG: 2/21/6, HC: 2/26/2).

A significant group (*F* = 23.3; *p* < 0.0001) and condition (*F* = 15.7; *p* < 0.001) effect was found for first step range of motion (**Table 2**). PD+FOG made significantly smaller first steps than PD-FOG who stepped shorter than HC in both the single and DT conditions (*p* < 0.01). PD+FOG and HC had significantly smaller first steps in the DT condition compared to the single task condition (*p* < 0.05). Significant group (*F* = 15.5; *p* < 0.0001) and condition (*F* = 113.9; *p* < 0.0001) effects were found for gait speed. PD+FOG and PD-FOG walked slower than HC (*p* < 0.01) and all groups walked slower in the DT condition compared to the single task condition (*p* < 0.0001). No significant group effects were found for any of the postural sway measures during 30 s of quiet stance prior to gait initiation (**Table 2**).

### Subjects with a History of FOG Showed More Hip Muscles Co-contraction than the Other Groups

In the single task condition, the groups differed significantly in the amount of co-contraction of the left and right TFL (*p* = 0.014), the amount of activity of the TFL of the stance leg (*p* = 0.015) and stepping leg (*p* = 0.029), and the duration of GAS activity of the stepping leg (*p* = 0.03) during trials without FOG episodes. *Post hoc* comparisons showed that PD+FOG had significantly more co-contraction of the left and right TFL (*p* = 0.005) and a longer duration of activity of the TFL of both the stance (*p* = 0.017) and stepping (*p* = 0.016) leg compared to PD-FOG (Supplementary Material).

In the DT condition, the groups significantly differed in the amount of co-contraction of GAS and TIB of the stance leg (*p* = 0.029) with PD+FOG having less ankle EMG activity than PD-FOG (*p* = 0.009) (Supplementary Material).

### Larger ML APAs Were Observed Prior to FOG Episodes

Two participants experienced FOG in the single task condition and seven subjects showed FOG in the DT condition. One participant was not able to make a step due to FOG in the DT condition and data from another subject could not be analyzed due to artifacts in the signal. The size of the averaged ML APAs in the trials with observed FOG episodes was 3 SD larger compared to the trials of PD+FOG who did not experience FOG during the assessment for both single and DT conditions (red circles in **Figure 2B**) and within the upper range of PD-FOG and HC.

### ML Size of APA Was Positively Associated with FOG Severity

The size of ML APAs was positively correlated with FOG severity, as measured with the NFOG-Q in the PD+FOG group (*p* = 0.025; Spearman’s Rho = 0.477, DT condition) (**Figure 2C**). In contrast, the size of ML APAs did not significantly correlate with the MDS-UPDRS III, PIGD, Mini-BESTest, MoCA, first step range of motion, gait speed, amount of co-contraction of left and right TFL, or any of the postural sway measures in either the PD+FOG or PD-FOG groups (*p* < 0.05). In all three groups (except HC DT condition), the ML size of APAs was significantly related to the first step time in both the single and DT conditions, showing larger ML size of APAs related to faster first steps.

## Discussion

This study investigated whether impaired weight shifting during the APA prior to a step contributes to the occurrence of start hesitation in PD with FOG. We compared APAs during self-initiated gait in individuals with PD with and without FOG and HC, with and without a concurrent, cognitive DT. Although PD subjects with FOG had smaller ML APAs than PD-FOG or controls in trials without start hesitation, their APAs were larger than the other two groups in the trials in which start hesitation was actually observed. Since large APAs were associated with more severe self-perceived FOG, we propose that the small APAs are not the cause of FOG, but a compensation to avoid FOG events.

### Subjects with a History of FOG Showed Reduced Size of APAs

An important finding of this study is that during self-initiated gait without start hesitation PD+FOG in the off medication state show reduced size of ML APAs compared to both PD-FOG and HC. It has been shown that the size of the APA is related to the displacement of the body center of mass during gait initiation (Breniere et al., 1981; Winter, 1995). Furthermore, in agreement with other studies (Delval et al., 2014; Plate et al., 2016) we did not find any group differences in the duration of APAs. Additionally, our groups did not differ in number of APAs. This indicates that the groups differently accelerated the center of mass during similar amount of time. We therefore suggest that the reduced ML size of APA in PD+FOG represents reduced lateral weight shifting when initiating gait. We found a higher amount of co-contraction of the left and right TFL during APA in PD+FOG which might explain the reduced ML size of APA.

The smaller size of ML APAs in PD+FOG compared to PD-FOG is neither a result of abnormal postural sway during quiet stance nor due to reduced gait speed. No group differences were found in postural sway prior to stepping and no association was found for size of ML APA and postural sway. Additionally, although PD+FOG had slower gait speed than PD-FOG and HC, no correlation was found for size of APA and gait speed in any group. These findings are consistent with distinct neural mechanisms for the generation of APAs and for postural control during quiet stance or gait speed (Schoneburg et al., 2013).

The size of ML APAs was significantly smaller in the PD+FOG than PD-FOG for the DT condition, but not the single-task condition. In addition, subjects with a history of FOG showed seven start hesitation events in the DT condition but only two start hesitation events in the single-task condition and more ankle muscle co-contraction was found in PD+FOG than PD-FOG only in the DT condition. These results suggest that the performance of gait initiation is more impaired when PD+FOG’s attention is focused on a cognitive task, consistent with less automatic step initiation in PD+FOG (Camicioli et al., 1998; Spildooren et al., 2010; de Souza Fortaleza et al., 2017). Thus, differences in gait initiation between PD+FOG and PD-FOG can be enhanced with a DT.

Other studies did not find any differences in ML size of APA when comparing PD+FOG with PD-FOG (Delval et al., 2014; Plate et al., 2016). In contrast to our study, these studies compared PD subjects in the on state of medication, which might explain different findings. Our groups were well-balanced concerning age and gender and PD+FOG and PD-FOG group differences cannot be explained by different severity of disease.

### Start Hesitation Is Not Caused by Small APAs

Although PD+FOG had smaller ML size of APA when successfully initiating gait, trials with FOG showed larger ML APAs which were within the upper range of HC and PD-FOG. In fact, we found a positive correlation between the size of ML APAs and the NFOG-Q, indicating that the more severe perceived FOG, the larger the size of ML APAs. Therefore, the hypothesis that start hesitation is caused by insufficient weight shifting when preparing gait is not supported by our data.

### Small APAs May Be a Compensatory Strategy for Individuals with FOG

One explanation for the altered APAs might be that the reduction of size of ML APAs is a compensatory strategy to successfully initiate gait in PD+FOG. This idea is supported by the positive correlation of the ML size of APA and FOG severity showing that the subjects with small, abnormal APAs most often successfully initiate gait whereas those subjects with larger APA which were within the range of HC most often experience FOG. The small APAs which represent reduced acceleration of the center of mass in PD+FOG might be a result of posture–gait coupling deficits in PD+FOG (Schlenstedt et al., 2016; Bekkers et al., 2017). PD+FOG might not be able to control large accelerations of the center of mass, which might cause a failure of coupling the APA with the stepping pattern. The move toward large APA during trials with FOG indicates a total break-down of the usual gait initiation pattern resulting in FOG. On the other hand, we could not detect any stereotyped pattern when analyzing the seven trials with start hesitation but found various different APA patterns. Furthermore, we found various amounts of muscular co-contraction during trials with observed FOG. This indicates that start hesitation cannot exclusively be explained by impaired ML weight shifting or muscular co-contraction but other mechanisms might play a role in the occurrence of gait initiation failure.

The following aspects should be considered when judging the validity of the experiment: The sample size was relatively large for this kind of assessment and PD+FOG and PD-FOG were well balanced with respect to age, disease duration, and disease severity. APAs were analyzed with a method as previously described and validated (Mancini et al., 2016) and each trial was plotted separately and visually checked to avoid artifacts. However, the following limitations have to be announced: The number of trials with observed FOG is small, not allowing statistical analysis. Additionally, although the participants were instructed to voluntarily start walking in a comfortable pace, the signal which indicated the end of the 30 s stance time might have impacted gait initiation as a cue.

In summary, this study shows that people with PD with a history of FOG have reduced ML APAs during gait initiation compared to PD-FOG and HC. Our data further show that start hesitation is not caused by insufficient weight shifting. Due to the positive correlation of ML size of APA and FOG severity we suggest that reducing the acceleration of the body center of mass might be a compensatory strategy in PD+FOG, addressing postural control deficits and enabling the subjects to successfully initiate stepping. Further research is necessary to investigate the role of the altered APA and FOG and to study which other mechanisms might co-exist contributing to the occurrence of start hesitation.

## Author Contributions

CS, MM, JN, and FH were involved in conception or design of the work. CS, MM, and AH analyzed the data. CS, MM, FH, WM, and GD performed data interpretation. CS drafted the work. MM performed the data acquisition. CS, MM, JN, FH, AH, WM, and GD revised the work critically for important intellectual content. All authors approved the final version of the paper to be published and agreed to be accountable for all aspects of the work in ensuring that questions related to the accuracy or integrity of any part of the work are appropriately investigated and resolved.

## Conflict of Interest Statement

FH has an equity/interest in APDM, a company that may have a commercial interest in the results of the study. This potential conflict of interest has been reviewed and managed by the Research & Development Committee at the Portland VA Medical Center and OHSU. The other authors declare that the research was conducted in the absence of any commercial or financial relationships that could be construed as a potential conflict of interest.

## Abbreviations

AP: anterior–posterior
FOG: freezing of gait
GAS: gastrocnemius medialis
H&Y: Hoehn and Yahr
HC: healthy control
MDS-UPDRS: movement disorders unified Parkinson’s disease rating scale
ML: medio-lateral
NFOG-Q: new freezing of gait questionnaire
PD+FOG: patients with FOG
PD-FOG: patients without FOG
PD: Parkinson’s disease
RMS: root mean square
TFL: tensor fasciae latae
TIB: tibialis anterior
