# Supplementary material for: Are Hypometric Anticipatory Postural Adjustments Contributing to Freezing of Gait in Parkinson’s Disease?
Source: Front Aging Neurosci. 2018 Feb 15;10:36. doi: 10.3389/fnagi.2018.00036 (PMC5818427; doi:10.3389/fnagi.2018.00036)
Supplement: Supplementary file 1 [file Data_Sheet_1.DOCX]

**Are Hypometric Anticipatory Postural Adjustments Contributing to Freezing of Gait in Parkinson's disease?**

**Supplemental Material Online**

Christian Schlenstedt^1,2^, Martina Mancini^2^, Jay Nutt^2^, Amie P. Hiller^2^, Walter Maetzler^1^, Günther Deuschl^1^, Fay Horak^2^

^1^ Department of Neurology, Christian-Albrechts-University, Kiel, Germany;

^2^ Balance Disorders Laboratory, Oregon Health & Science University, Portland, Oregon, USA

Figure 1. Muscle activity during APA.


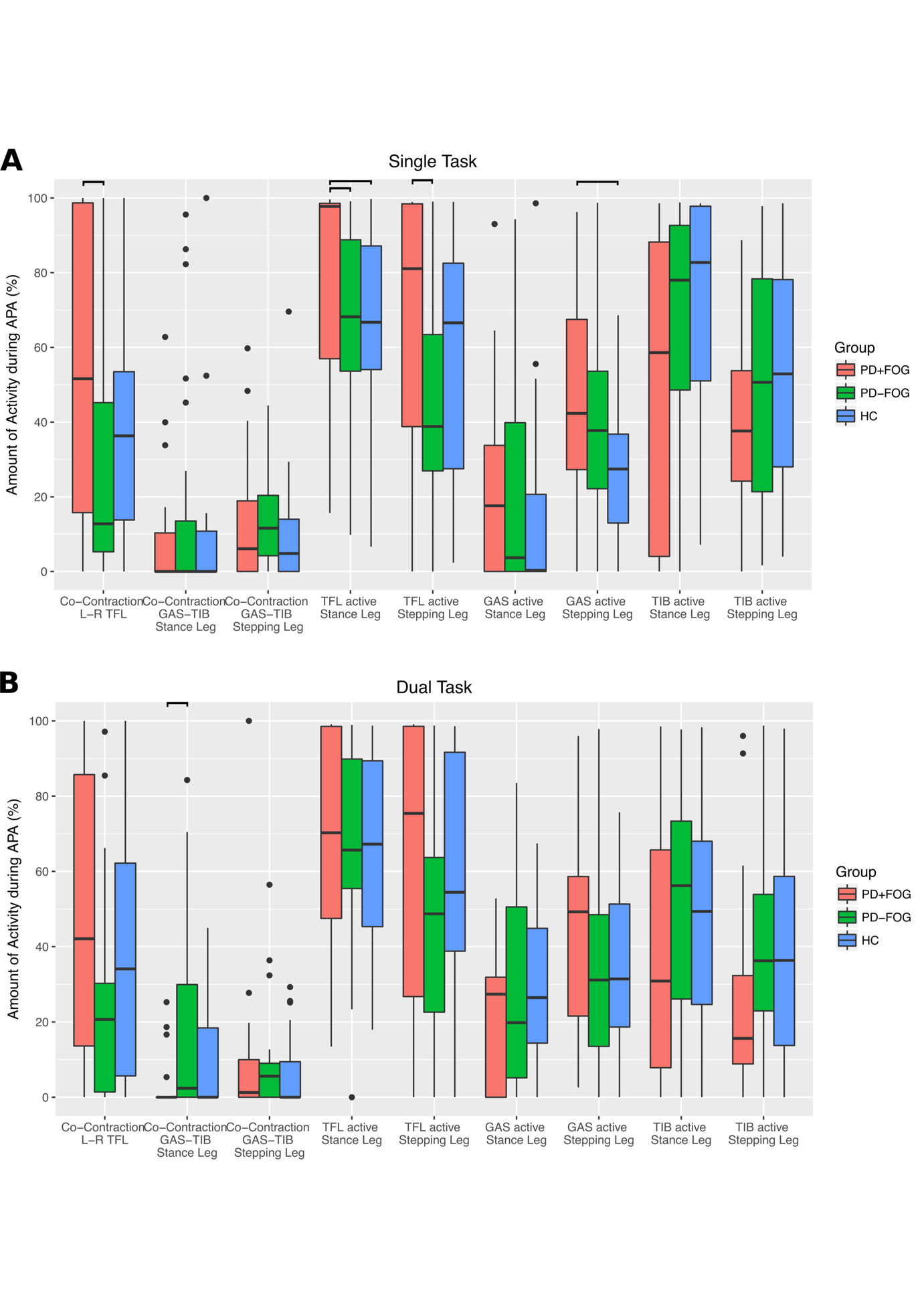


Table. Muscle activity during APA.

| Variable | Condition | PD+FOG | PD-FOG | HC | p-value^#^ |
| --- | --- | --- | --- | --- | --- |
| Co-contraction TFL during APA (%) | Single Task | 51.6 (0-100)^a^ | 12.7 (0-100)^a^ | 36.3 (0-100) | 0.014* |
|  | Dual Task | 42.1 (0-100) | 20.7 (0-97.1) | 34.1 (0-100) | 0.139 |
| Co-contraction GAS and TIB of Stance Leg during APA (%) | Single Task | 0 (0-62.8) | 0 (0-95.6) | 0 (0-100) | 0.948 |
|  | Dual Task | 0 (0-25.3)^b^ | 2.4 (0-84.3)^b^ | 0 (0-45.0) | 0.029* |
| Co-contraction GAS and TIB of Stepping Leg during APA (%) | Single Task | 6.1 (0-59.7) | 11.6 (0-44.4) | 4.8 (0-69.6) | 0.17 |
|  | Dual Task | 1.2 (0-100) | 5.6 (0-56.5) | 0 (0-29.3) | 0.56 |
| TFL of Stance Leg active during APA (%) | Single Task | 97.7 (15.6-99.6)^c,d^ | 68.2 (9.8-99.1)^c^ | 66.7 (6.6-99.7)^d^ | 0.015* |
|  | Dual Task | 70.3 (13.5-99.1) | 65.7 (0-98.9) | 67.2 (17.9-98.7) | 0.535 |
| TFL of Stepping Leg active during APA (%) | Single Task | 81.1 (0-98.8)^e^ | 38.8 (0-99.0)^e^ | 66.6 (2.4-98.9) | 0.029* |
|  | Dual Task | 75.4 (0-99.1) | 48.7 (0-98.7) | 54.4 (0-98.6) | 0.16 |
| GAS of Stance Leg active during APA (%) | Single Task | 17.6 (0-93.0) | 3.7 (0-94.3) | 0.3 (0-98.6)^f^ | 0.414 |
|  | Dual Task | 27.4 (0-52.8) | 19.8 (0-83.5) | 26.5 (0-67.4)^f^ | 0.522 |
| GAS of Stepping Leg active during APA (%) | Single Task | 42.3 (0-96.2)^g^ | 37.7 (0-98.7) | 27.4 (0-68.6)^g^ | 0.03* |
|  | Dual Task | 49.3 (2.6-96.0) | 31.2 (0-97.8) | 31.4 (0-75.7) | 0.371 |
| TIB of Stance Leg active during APA (%) | Single Task | 58.6 (0-98.6) | 78.0 (0-98.8)^h^ | 82.7 (7.1-98.6)^i^ | 0.116 |
|  | Dual Task | 30.9 (0-98.5) | 56.2 (0-97.7)^h^ | 49.4 (0-98.3)^i^ | 0.269 |
| TIB of Stepping Leg active during APA (%) | Single Task | 37.6 (0-88.7) | 50.7 (1.6-97.8) | 52.9 (4.0-98.6) | 0.209 |
|  | Dual Task | 15.6 (0-96.0) | 36.2 (0-98.8) | 36.4 (0-97.9) | 0.095 |

Note. Values represent median (range). ^#^ *p*-value of Kruskal-Wallis-Test; * significantly different (p<0.05); significant post-hoc comparisons (Holm-Bonferroni adjusted for multiple comparisons): a,b,d,h,f: p<0.01; c,e,g: p<0.05; i: p=0.0002.
